# A MEMS Variable Optical Attenuator with Ultra-Low Wavelength-Dependent Loss and Polarization-Dependent Loss

**DOI:** 10.3390/mi9120632

**Published:** 2018-11-29

**Authors:** Huangqingbo Sun, Wei Zhou, Zijing Zhang, Zhujun Wan

**Affiliations:** 1 School of Optical and Electronic Information, Huazhong University of Science and Technology, Wuhan 430074, China; u201514056@hust.edu.cn (H.S.); zijing_zhang@hust.edu.cn (Z.Z.); 2 AOFSS (Shenzhen) Co., Ltd., Shenzhen 518103, China; david.zhou@aofss.com; 3 Shenzhen Huazhong University of Science and Technology Research Institute, Shenzhen 518000, China

**Keywords:** variable optical attenuator (VOA), wavelength dependent loss (WDL), polarization dependent loss (PDL), micro-electro-mechanical systems (MEMS)

## Abstract

Applications in broadband optical fiber communication system need variable optical attenuators (VOAs) with low wavelength-dependent loss (WDL). Based on analysis on the dispersion of the optical system of a MEMS-based VOA, we provide a method to reduce the WDL significantly with minor revision on the end-face angle of the collimating lens. Two samples are assembled, and the measured WDL is <0.4 dB over the C-band (1.53–1.57 μm) at a 0–20 dB attenuation range. Meanwhile, the new structure helps to reduce the polarization-dependent loss (PDL) to <0.15 dB, which is only half that of conventional devices.

## 1. Introduction

A variable optical attenuator (VOA) is an important optical device for optical fiber communication and optical instrumentation [1,2]. The main approaches for a VOA include: thermo-optically adjusted Mach–Zehnder interferometer (MZI) based on a planar lightwave circuit (PLC) [3], optical fluid driven by a pump [4,5], liquid-core fiber driven by thermo-optical effect [6], and MEMS technology. Among the variable technologies, MEMS technology is one of the most favorable approaches for a VOA. Chengkuo Lee’s group did much work to develop different mechanisms for MEMS VOAs, such as retro-reflective mirrors driven by electro-thermal actuators [7], and reflective mirrors driven by rotary comb drive actuators [8]. The most applicable mechanisms are the MEMS shutter [9,10] and the MEMS torsion mirror [11].

One application of VOAs is in erbium-doped fiber amplifier (EDFA) modules for optical fiber communication. An EDFA usually amplifies broadband optical signals over 1.53–1.57 μm. The same amplification for different wavelengths is required. A VOA is employed in the EDFA module to control the optical power dynamically, which requires it to generate nearly the same attenuation for different wavelengths over the bandwidth of 40 nm. However, the existence of wavelength-dependent loss (WDL) means that the VOA generates different attenuation for different wavelengths. The MEMS torsion mirror, driven by comb drive actuators is characterized by low power consumption and ease of packaging, which enable a VOA to be created with low voltage, small size, and low cost. Thus, VOAs based on a MEMS torsion mirror are widely employed in optical fiber communication. However, they are confounded by high WDLs, especially when operating at a high attenuation level. The conventional VOAs without optimization usually show WDLs of more than 1 dB. Reducing the WDL helps to improve the specifications of the EDFA modules.

Researchers from Mega Sense Inc. presented an optimization method by axially rotating the lens with respect to the fibers, while the process is rather time-consuming [12]. Researchers from JDS Uniphase Corp. optimized the WDL by introducing a wedge prism, which added to the complexity of the device [13]. Researchers from NeoPhotonics Corp. analyzed the cause of WDL extensively, and provided an optimization method, while it requires the collimating lens to be fabricated with high dispersion glass [14].

For applications such as optical instrumentation, PDL is another important parameter for VOAs. The existence of PDL means that the VOA generates different attenuations for different polarizations of light. Reducing PDL helps to improve the precision of the optical instruments. PDL is usually introduced by angular surfaces (which are necessary for reduction of back-reflection) and stress in the optical system. The proposal in [14] requires that the angle of the collimating lens is more than 10 degrees, which will introduce more PDL.

Based on the work in [13,14], this paper systematically addressed the WDL and PDL problems in a VOA based on a MEMS torsion mirror. A simple solution for both WDL and PDL optimization was presented with no excess optical element and special glass material required.

## 2. Theories

### 2.1. Structure of the VOA

**Figure 1:**
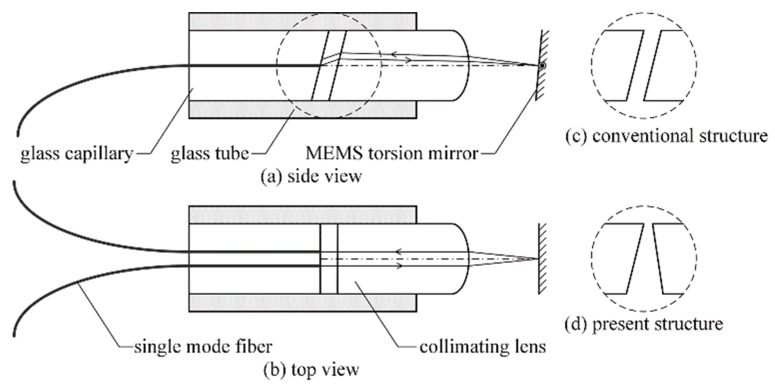
Structure of the variable optical attenuator (VOA) based on a MEMS torsion mirror.

The addressed VOA comprises a dual-fiber collimator and a MEMS torsion mirror coaxially assembled, as shown in Figure 1. The collimator includes a plano-convex collimating lens and two single-mode fibers (SMFs) fixed by a glass capillary. Both the collimating lens and the glass capillary are housed by a glass tube. The fiber facet and the MEMS torsion mirror are located at the front and rear focal planes of the collimating lens, respectively, as shown in Figure 1a,b. The optical signal is input from one optical fiber, and then collimated. The collimated beam is reflected by the mirror and then refocused onto the facet of another optical fiber. The refocused beam spot deviates from the output fiber core, due to the deflection of the mirror, and thus results in the desired attenuation of optical power. In order to reduce back reflection, the facet of the fibers and the planar facet of the lens are both angularly polished. For a conventional structure, both facets are usually polished with an angle of 8˚ and aligned parallel, as shown in Figure 1c. Based on analysis on the dispersion of the optical system, we provide a new design to optimize the WDL of the VOA. The facet of the fibers keeps unchanged, while the facet of the lens is polished with an angle of −7°. The two facets are aligned as Figure 1d. Meanwhile, the length of the lens is also adjusted, and the theories are shown in Section 2.2.

The optical attenuation is tunable by adjusting the tilt angle of the mirror, which results in a lateral offset *X* of the refocused beam spot on the facet of the output fiber. The attenuation due to lateral offset can be described as [13,15]:

(1) $$A=4.34{\left(\frac{X}{\omega }\right)}^{2}$$

where *ω* is the mode radius of the SMF.

### 2.2. WDL Analysis and Optimization

The mode size *ω*(*λ*) of the SMF is wavelength-dependent, and thus results in a wavelength-dependent attenuation *A*(*λ*) (which is usually called WDL) according to Equation (1). The wavelength dependence of the mode size is rather complicated. However, within a relatively narrow range, such as C-band, the mode size can be linearly approximated. Thus we can assume the mode radii as *ω_s_* = *ω_c_* − Δ*ω* and *ω_l_* = *ω_c_* + Δ*ω* for the shortest wavelength *λ_s_* and longest wavelength *λ_l_*, respectively, given *ω_c_* as the mode radius of the central wavelength *λ_c_*. According to Equation (1), the WDL before optimization can be obtained as:

(2) $$\mathrm{WDL}=4.34X^{2}\left(\frac{1}{\omega_{s}^{2}}-\frac{1}{\omega_{l}^{2}}\right)=A_{c}\omega_{c}^{2}\left(\frac{1}{\omega_{s}^{2}}-\frac{1}{\omega_{l}^{2}}\right)$$

which is the attenuation difference between *λ_l_* and *λ_s_* in the C-band. *A_c_* is the attenuation for wavelength *λ_c_*. Equation (2) shows that the WDL adds up when the attenuation level increases. For a VOA operating in the C-band (1.53–1.57 μm), the WDL is usually more than 1 dB at 20 dB attenuation.

According to Equation (1), if we can introduce a wavelength-dependent variable *X*, then it is possible to reduce the WDL. Considering the dispersion of *X*, the WDL can be rewritten as:

(3) $$\mathrm{WDL}=4.34\left[{\left(\frac{X_{c}+\Delta X}{\omega_{c}+\Delta \omega }\right)}^{2}-{\left(\frac{X_{c}-\Delta X}{\omega_{c}-\Delta \omega }\right)}^{2}\right]$$

We assume a linear dispersion for *X*, with *X_c_*, *X_c_* + Δ*X* and *X_c_* − Δ*X* as the lateral offset of the *λ_c_*, *λ_l_* and *λ_s_* beam spots, respectively.

According to Equation (3), if Equation (4) is satisfied, the WDL can be reduced to zero for a specified *X_c_*, which corresponds to a certain attenuation level *A_c_*. As shown in Figure 7 of [14], when a specific *A_c_* is selected for zero WDL design, the maximum WDL over the attenuation range (such as 0–20 dB) can be minimized. Here we choose *A_c_* = 13.3 dB for optimization design.

(4) $$\frac{\Delta X}{X_{c}}=\frac{\Delta \omega }{\omega_{c}}$$

**Figure 2:**
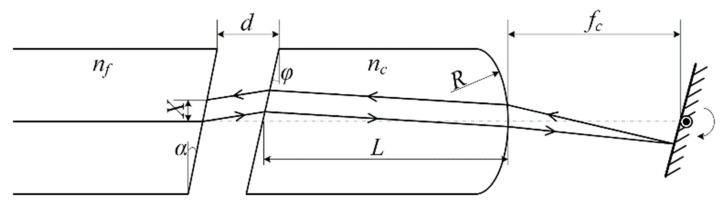
Optical model of the VOA in side view.

The dispersion Δ*X* of the lateral offset results from the dispersion of the optical system. The optical model of the VOA in side view is shown in Figure 2. *n_f_* and *n_c_* are the refractive indexes of the fiber core and the collimating lens, respectively. The focal length of the lens is *f_c_* = *R*/(*n_c_* − 1), where *R* is the curvature radius of the right surface. The fiber facet locates at the front focal plane of the collimating lens, and thus, the gap is obtained as *d* = *f_c_* − *L*/*n_c_*, where *L* is the length of the lens. The polished angles of the optical fiber and the collimating lens are *α* and *φ*, respectively. The deflection of the mirror results in offset *X* of the beam spot, focused on the output fiber facet. Considering the dispersion effect, the subscript *c* in *n_c_* and *f_c_* is the parameter corresponding to the central wavelength *λ_c_*.

Based on the above optical model, the lateral offset *X* and the dispersion Δ*X* of the optical system are obtained as Equations (5) and (6) after ray tracing and paraxial approximation:

(5) $$X=X_{c}+2\Delta n\left[d\varphi +\frac{d\beta }{n_{c}-1}+\frac{\beta L-(n_{c}-1)\varphi L}{n_{c}^{2}(n_{c}-1)}\right]$$

(6) $$\Delta X=2\Delta n\left[d\varphi +\frac{d\beta }{n_{c}-1}+\frac{\beta L-(n_{c}-1)\varphi L}{n_{c}^{2}(n_{c}-1)}\right]$$

where *β* = (*n_f_* − 1)*α*, and Δ*n* = *ns* − *nc* = *nc* − *nl* (the subscripts *s* and *l* correspond to the shortest and longest wavelengths in the C-band, respectively) is the difference on refractive index of the lens. By substituting Equation (6) into Equation (4), we obtain Equation (7) as follows,

(7) $$d\varphi +\frac{d\beta }{n_{c}-1}+\frac{\beta L-(n_{c}-1)\varphi L}{n_{c}^{2}(n_{c}-1)}=\frac{\Delta \omega X_{c}}{2\Delta n\omega_{c}}$$

The fiber employed is SMF-28 by Corning Corp. (Corning, NY, USA) and the glass for the collimating lens is N-SF11 by Schott Corp. (Mainz, Germany). The given parameters are *n_f_* = 1.4682, Δ*ω* = 0.0622 μm, *α* = 8° and *n_c_* = 1.7434, Δ*n* = 0.00036, *R* = 1.419 mm. Thus there are only two parameters related by Equation (7), i.e., the facet angle *φ* and the length *L* of the lens.

**Figure 3:**
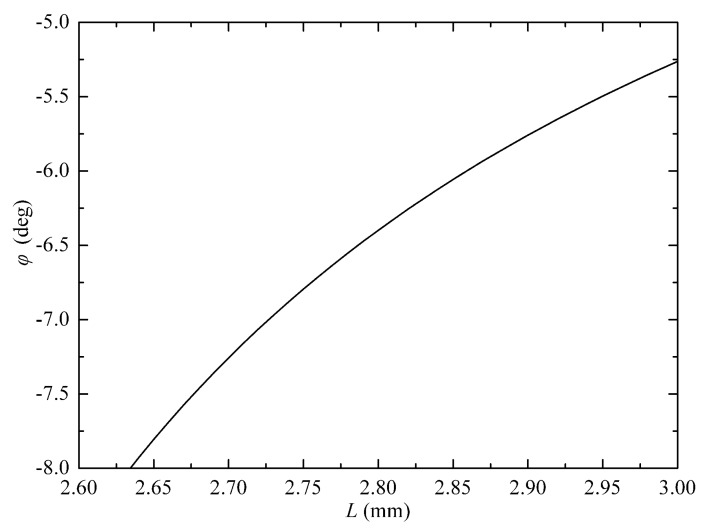
Correlation between the parameters of the collimating lens.

Equation (7) is rather complicated. The correlation between *φ* and *L* is numerically plotted, as shown in Figure 3. The lens length is *L* = *n_c_*(*f_c_* − *d*) < *n_c_f_c_* = 3.33 mm. Thus, we just plot the curve with the range of *L* being 2.6–3.0 mm. Parameters corresponding to any point on the curve can be employed for WDL optimization.

### 2.3. Return Loss Consideration

**Figure 4:**
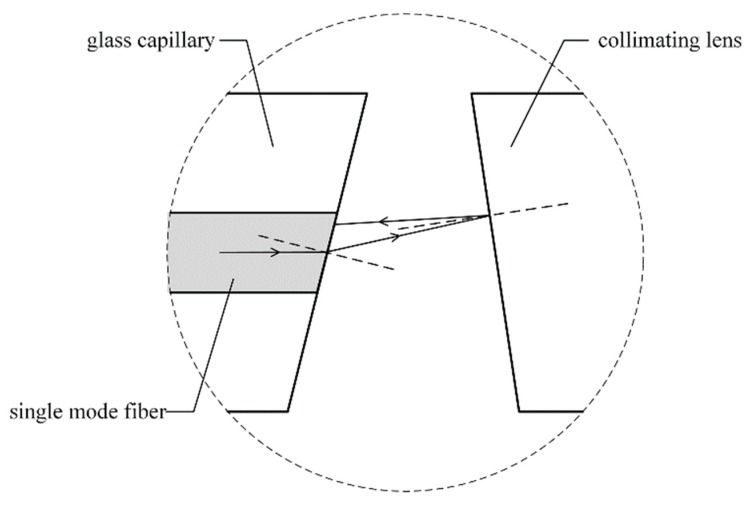
Back flection from the left facet of the collimating lens.

In order to reduce the back reflection, the facets of the optical fibers and the collimating lens are usually angled, polished, and aligned, as in Figure 1c. However, we find in Figure 3 that the angle *φ* must be negative, which means that the facets of the optical fibers and the collimating lens are aligned as in Figure 1d. Thus, the reflected light from the left facet of the lens is more likely to return to the optical fiber than the conventional structure, as shown in Figure 4.

The return loss (*RL*) can be obtained based on fiber-to-fiber coupling with angular and longitudinal misalignments. By simplifying Equation (33) in [15] with zero lateral misalignment, the *RL* is obtained as Equation (8), with consideration of the angular misalignment *θ* and the longitudinal misalignment *Z*_0_, as well as the residual reflection *R_r_* at the AR (anti-reflection)-coated left facet of the lens:

(8) $$RL=-10\log\left(R_{r}\right)-10\log\left\{\frac{4}{C^{2}+4}\exp\left[-\frac{kZ_{0}\left(C^{2}+2\right)\sin^{2}\theta }{C\left(C^{2}+4\right)}\right]\right\}$$

where *C = λ_c_Z*_0_/*πω_c_*^2^. Based on ray tracing and paraxial approximation, the angular and longitudinal misalignments are obtained as *θ =* −2(*φ* + *β*) and *Z*_0_ = 2*d (d = f_c_* − *L*/*n_c_)*, respectively. Note that all of the surfaces in the optical system are AR-coated. The reflection from the other facets are negligible.

According to Equation (8), the *RL* is improved when ǀ*φ*ǀ increases and *L* decreases. We choose a point from the curve in Figure 3. The corresponding parameters are *φ* = −7° and *L* = 2.72 mm. The residual reflection from the lens facet is *R_r_* = 0.1% (by sample measurement). Thus, *RL* is calculated as 53 dB, according to Equation (8).

### 2.4. PDL Analysis

PDL is usually introduced by angular surfaces and stress in the optical system. The influence of stress depends on the materials and the assembly process, which is outside the scope of this paper. We focus on the influence of the angled surfaces.

Because all of the surfaces in the optical system are AR-coated, the difference between the p-ray and s-ray transmittance introduced by the angular surfaces is negligible. The angular surfaces expand or compress the optical beam only in the tangential plane, and thus result in a slightly elliptical beam spot focused on the facet of the output fiber. When the elliptical beam is received by the circular optical fiber, PDL is introduced.

A circular beam emits from the input fiber, and finally an elliptical beam is focused on the facet of the output fiber. The transformation can be obtained by tracing of the Gaussian beam. Gaussian beam tracing is based on q-parameter and ABCD matrices of the optical elements. When a Gaussian beam is refracted by an angular facet tilted in tangential plane, the ABCD matrix shows different forms in tangential and sagittal planes as Equations (9) and (10), respectively [16].

(9) $$M_{T}=\left(\begin{array}{cc} \frac{\cos\theta_{2}}{\cos\theta_{1}} & 0\\ 0 & \frac{n_{1}\cos\theta_{1}}{n_{2}\cos\theta_{2}} \end{array}\right)$$

(10) $$M_{S}=\left(\begin{array}{cc} 1 & 0\\ 0 & \frac{n_{1}}{n_{2}} \end{array}\right)$$

where *θ*_1_ and *θ*_2_ are the incidence and refraction angles, respectively, and *n*_1_ and *n*_2_ are the refractive indices of the materials before and after the angular surface, respectively. Based on Gaussian beam tracing through the entire optical system, the radii of the beam focused on the output fiber facet is obtained. For the conventional structure, the sizes are *ω_T_* = 5.184 μm and *ω_S_* = 5.224 μm (note that the subscripts *T* and *S* correspond to the tangential and sagittal planes, respectively), with a difference of Δ*ω_TS_* = 0.04 μm. For the present structure, the sizes are *ω_T_* = 5.184 μm and *ω_S_* = 5.187 μm, with a difference of Δ*ω_TS_* = 0.003 μm. The ellipticity is reduced to 1/13, and thus the PDL can be optimized.

## 3. Experimental Results

Based on above analysis, we designed a MEMS VOA operating at 1.53–1.57 μm. The type of the input/output optical fibers is SMF-28 by Corning Inc. (Corning, NY, USA), with related parameters summarized in Table 1. We keep the facet angle of the optical fiber as 8°, which is the same as the conventional structures. The collimating lens is designed according to the optimization curve in Figure 3. The lens material is N-SF11 by Schott Inc. (Mainz, Germany), with parameters summarized in Table 2. The MEMS mirror is provided by Preciseley Microtechnology Corp. (Edmonton, AB, Canada), and the parameters are summarized in Table 3.

**Figure 5:**
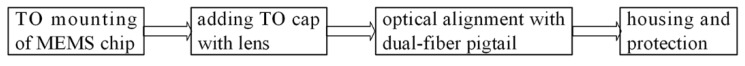
Schematic diagram of the assembling procedures.

**Figure 6:**
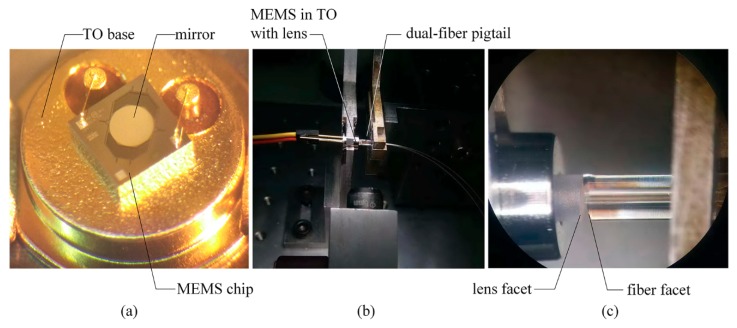
Alignment and assembly of the MEMS VOA. (**a**) MEMS chip assembled on a TO base, (**b**) optical alignment of the VOA, (**c**) enlarged view between the lens and fiber facets.

**Figure 7:**
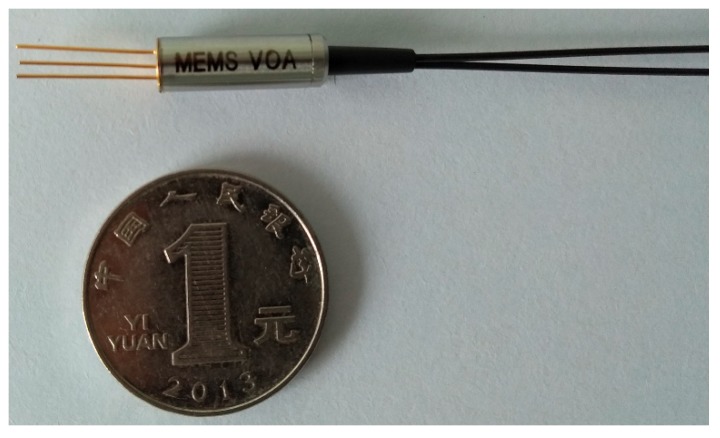
Photograph of the VOA sample.

Figure 5 shows the schematic diagram of the assembly procedures. The MEMS chip was first mounted on a Transister Outline (TO) base, as shown in Figure 6a. Then, a specially designed TO cap was added, with the collimating lens mounted in the cap. Finally, the sub-assembly of MEMS in TO with the lens was aligned with a dual-fiber pigtail by mechanical stages, as shown in Figure 6b. The two parts were fixed with adhesive after optical alignment. According to the above design, the facets of the lens and fiber were aligned as Figure 1d. In real assembly, the enlarged view between the lens and fiber facets is shown in Figure 6c. The final assembly of the MEMS VOA is shown in Figure 7, with the MEMS and optical parts protected by a metal housing and the pigtail fibers protected by plastic tubes of Ф0.9 mm. The samples were assembled in AOFSS (Shenzhen) Ltd., Shenzhen, China, and the product type is LMA-1 B20LV1L1000.

The measured specifications of two samples are summarized in Table 4, including the insertion loss (IL), *RL*, and PDL at different attenuation levels. For comparison, a conventional MEMS VOA assembled as in Figure 1c was also measured, and the specifications were also summarized in Table 4. As we can see, the new samples show specifications of IL < 0.6 dB, *RL* > 50 dB, and PDL < 0.15 dB over the attenuation range of 0–20 dB, which meet the application requirements. The PDL is reduced to nearly half of the conventional VOA. Meantime, the IL is increased by 0.14 dB, and the *RL* is reduced by 4.8 dB.

**Figure 8:**
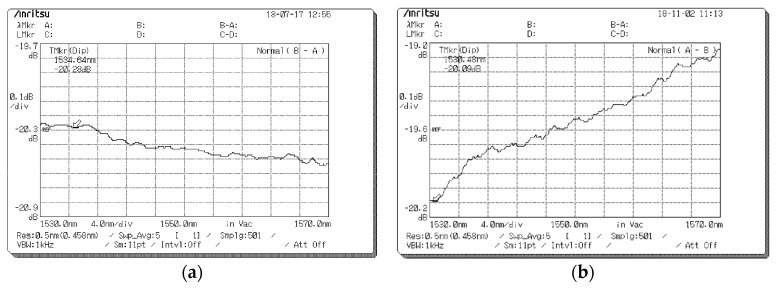
Measured transmission spectrum at an attenuation level of 20 dB, (**a**) the optimized MEMS VOA, (**b**) the conventional MEMS VOA.

In order to obtain the WDL specifications, the spectra of the new samples and the conventional VOA were measured with an optical spectrum analyzer. Figure 8a and Figure 8b show the spectra of sample #1 and the conventional one at the attenuation level of *A_c_* = 20 dB, respectively. For the former one, the WDL over the C-band @20 dB can be read as 0.28 dB from the spectrum curve, while the latter one is read as 1.07 dB.

**Figure 9:**
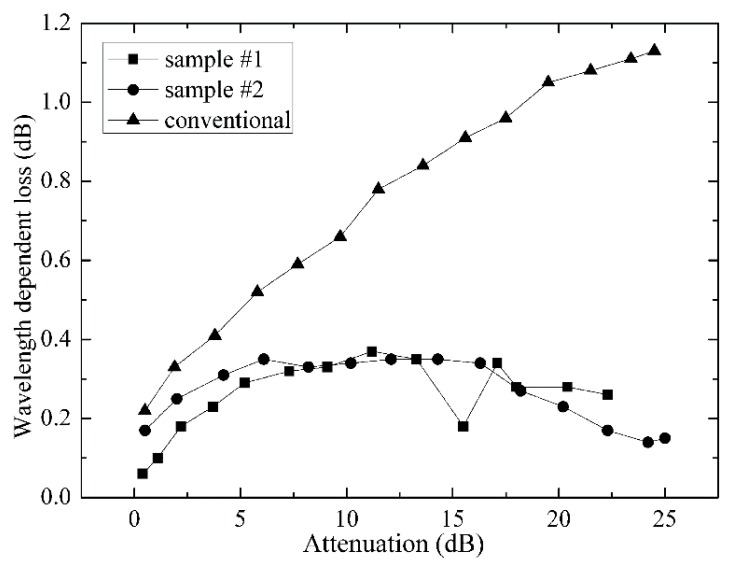
Measured WDL over the C-band at different attenuation levels.

The measured WDL over the C-band at different attenuation levels is summarized in Figure 9. The two new samples show a maximum WDL of <0.4 dB, while the maximum WDL of the conventional VOA is 1.07 dB at 20 dB attenuation. The new design reduces the WDL significantly.

## 4. Conclusions

We addressed the WDL and PDL problems in a VOA, based on a MEMS torsion mirror. The causes for WDL and PDL were analyzed, and a simple optimization method was presented with the consideration of return loss. The experimental results show that the maximum WDL is <0.4 dB over the wavelength range 1.53–1.57 μm @0–20 dB attenuation range, and the maximum PDL at a 0–20 dB attenuation range is reduced to <0.15 dB, which is only half that of the conventional devices. The results verify the effectiveness of the optimization method well.

## Figures and Tables

**Table 1:** Parameters of the optical fiber.

Optical Fiber	Refractive Index	Mode Radius	Mode Dispersion	Facet Angle
Corning SMF-28	*n_f_* = 1.4682	*ω_c_* = 5.2 μm	Δ*ω* = 0.0622 μm	*α* = 8˚

**Table 2:** Parameters of the collimating lens.

Material	Refractive Index	Length	Curvature Radius	Facet Angle
Schott N-SF11	*n_c_* = 1.7434	*L* = 2.72 mm	*R* = 1.419 mm	*φ* = −7°

**Table 3:** Parameters of the MEMS mirror.

MEMS Mirror	Clear Aperture	Maximum Tilting Angle
Preciseley LV-VOA	Ф0.85 mm	0.35° @5 V

**Table 4:** Measured specifications of the new samples and a conventional MEMS VOA.

No.	IL	*RL*	PDL (dB)				
	(dB)	(dB)	@IL	@5 dB	@10 dB	@15 dB	@20 dB
#1	0.55	52.2	0.02	0.05	0.07	0.10	0.10
#2	0.58	51.5	0.01	0.04	0.05	0.07	0.12
Conventional	0.44	56.3	0.04	0.07	0.13	0.19	0.26
